# Comparative Analysis of Differentially Mutated Genes in Non-Muscle and Muscle-Invasive Bladder Cancer in the Chinese Population by Whole Exome Sequencing

**DOI:** 10.3389/fgene.2022.831146

**Published:** 2022-03-25

**Authors:** Fangming Wang, Xiying Dong, Feiya Yang, Nianzeng Xing

**Affiliations:** ^1^ State Key Laboratory of Molecular Oncology, National Cancer Center/National Clinical Research Center for Cancer/Cancer Hospital, Chinese Academy of Medical Sciences and Peking Union Medical College, Beijing, China; ^2^ Department of Urology, National Cancer Center/National Clinical Research Center for Cancer/Cancer Hospital, Chinese Academy of Medical Sciences and Peking Union Medical College, Beijing, China; ^3^ Peking Union Medical College Hospital, Peking Union Medical College and Chinese Academy of Medical Sciences, Beijing, China; ^4^ Department of Urology, Shanxi Province Cancer Hospital/Shanxi Hospital Affiliated to Cancer Hospital, Chinese Academy of Medical Sciences/Cancer Hospital Affiliated to Shanxi Medical University, Taiyuan, China

**Keywords:** mutated gene, muscle invasive bladder cancer, non-muscle invasive bladder cancer, whole exome sequencing, comparative analysis

## Abstract

**Objective:** To characterize the spectra of mutations in non-muscle invasive bladder cancer (NMIBC) and muscle-invasive bladder cancer (MIBC) in the Chinese population to identify any mutational features and find potential therapeutic targets.

**Materials and methods:** We collected fresh bladder tumor samples from NMIBC (n = 9) and MIBC patients (n = 11) along with adjacent normal bladder tissue specimen and peripheral blood sample. Using whole exome sequencing (WES), we analyzed the mutation spectra of those NMIBC and MIBC bladder cancer (BCa) specimen.

**Results:** Our results demonstrated that 95% of BCa patients (19/20) had varying degrees of driver gene mutations, FGFR3 (45%), KMT2D (40%), PIK3CA (35%), ARID1A (20%), EP300 (20%), KDM6A (20%), KMT2C (20%), and STAG2 (20%) were the most frequently mutated genes in BCa patients. NMIBC and MIBC exhibited different genomic alterations. FGFR3 (67%), PIK3CA (56%), and RHOB (44%) were the most frequently mutated genes in NMIBC patients. Of note, RHOB mutation only occurred in NMIBC, whereas mutations of KMT2D (55%), TP53 (36%) and KMT2B (27%) were frequently detected in MIBC, and TP53 and KMT2B mutation only occurred in MIBC. The frequency of mutations in DNA-damage repair (DDR) gene was higher in MIBC than that in NMIBC (91 vs 78%, 6.2 vs 2.4 gene mutations per patient). Copy number alterations (CNAs) occurred at more diverse chromosomal locations in NMIBC, but the CNA burden was higher in MIBC [9.01 (2.07–31.51) vs 4.98 (0.99–9.73) mutations/Mb]., the trend of which was consistent with the tumor mutation burden (TMB) [8.26 (4.63–21.84) vs 5.58 (3.87–9.58) mutations/Mb]. Among the current set of single-base substitution (SBS) signatures including SBS 1, 2, 5, 13, and 40, we identified one differently expressed signature between NMIBC and MIBC patients: SBS13.

**Conclusions:** There were different gene mutational characteristics and signatures between NMIBC and MIBC in the Chinese population. Frequency of DDR, CNA burden and TMB were higher in MIBC. Our analysis revealed that several genes in NMIBC did not overlap with those reported in MIBC, suggesting that a fraction of NMIBC and MIBC likely developed secondary to different precursor lesions.

## Introduction

Bladder cancer (BCa) is the most common malignancy of the urinary tract, which occurred in 573, 278 patients (5.6/100,000) and caused 212, 536 deaths globally in 2020, accounting for 3.0 and 2.1% of the total cancer incidence and mortality secondary to malignancy, respectively (Sung et al., 2021). In China, the overall incidence and mortality of BCa were about 5.8/100,000 and 2.37/100,000 in 2015 and continued to increase in the recent years (Chen et al., 2015). Urothelial (formerly transitional cell) carcinoma accounted for approximately 90% of all BCa (Babjuk et al., 2017).

BCa was divided into non-muscle invasive bladder cancer (NMIBC) (stage Ta or T1) and muscle-invasive bladder cancer (MIBC) (stage T2, T3, or T4) according to pathologic tumor staging which was based upon whether invasion into muscle was present. NMIBC accounted for 70–80% of BCa both in China and Europe (Li et al., 2015; Babjuk et al., 2017). In clinical practice, patients with NMIBCs were treated with transurethral resection of the bladder tumor (TURBT) followed by an intravesical instillation of chemotherapy or *Bacillus* Calmette-Guerin (BCG) therapy. Nevertheless, half of them can relapse and 5–20% of NMIBC will eventually progress to MIBC despite of all the treatments (van den Bosch and Alfred Witjes, 2011; Jordan and Meeks, 2019). MIBC patients, who were treated with a systemic cisplatin-based neoadjuvant chemotherapy followed by a radical cystectomy, have a poor prognosis with less than 50% 5-year survival (Alfred Witjes et al., 2017). Therefore, there was an urgent need to clarify the related molecular tumorigenic mechanisms and develop novel targeted therapies of MIBC and NMIBC.

Unlike many other types of cancer, NMIBC does not always advance to MIBC. However, when this occurs, the prognosis is even worse. This indicates that NMIBC and MIBC have different molecular characteristics and were largely believed to develop secondary to different molecular alterations, though there could be connections between them (Audenet et al., 2018; Cao et al., 2020). Historically, NMIBC was considered to evolve from epithelial hyperplasia, whereas MIBC arose from dysplasia and was associated with genetic instability (Jones and Droller, 1993).

In the recent years, next generation sequencing (NGS) has been applied to analyze genomic alterations in BCa. BCa has been found to exhibit a high frequency of somatic mutations compared to other solid tumors (Knowles and Hurst, 2015; Pietzak et al., 2017). Mutation of FGFR3 was identified as a driver mutation of NMIBC (Al Hussain and Akhtar, 2013), while most of MIBC are characterized by inactivating mutations involving major tumor suppressors such as TP53, RB1, and PTEN (Cordon-Cardo, 2008). Recently, there have been large-scale analyses that comprehensively investigated the genes mutated in NMIBC and MIBC by analyzing The Cancer Genome Atlas (TCGA) database or performing NGS by other groups (The Cancer Genome Atlas Research Network, 2014; Pietzak et al., 2017; Robertson et al., 2017). However, the molecular profile and mutational characteristics of NMIBC and MIBC at the genomic level in Chinese Han population have not been extensively investigated. As far as we know, there were only five studies investigating BCa genomic alterations in the Chinese population (Gui et al., 2011; Guo et al., 2013; Pan et al., 2016; Wu et al., 2019; Wang et al., 2020), and none of them used WES instead of targeted NGS to systemically compare the characteristics of gene mutations in NMIBC and MIBC. In this study, using WES, we comprehensively examined, analyzed and compared the mutation spectra of nine NMIBC and 11 MIBC cases, including the frequencies of DNA-damage repair (DDR), tumor mutation burden (TMB), copy number alterations (CNA), and signatures. Our study found genetic mutations unique to NMIBC or MIBC, which may help to explore the pathogenesis and mechanisms, and provide potential biomarkers and novel treatment targets for BCa.

## Materials and Methods

### Patients and Samples

Fresh clinical BCa tissues were collected in accordance with the Declaration of Helsinki 1975) and was approved by the ethical committee of National Cancer Center. All patient names, initials, or hospital numbers were not demonstrated in this study. The study was conducted in patients with pathologically confirmed bladder urothelial carcinoma, between January 2021 and June 2021 at the Department of Urology at the National Cancer Center/National Clinical Research Center for Cancer/Cancer Hospital. All patients were Chinese Han people. The tumor staging was assessed according to the Union Internationale Contre le Cancer (UICC) TNM classification of malignant tumors 2017. And the grade was assessed according to the WHO classification of 2004. A total of nine fresh BCa tissues and their corresponding blood samples were collected from NMIBC patients who underwent TURBT, while 11 fresh BCa tissues and their respective non-neoplastic bladder specimen were collected from MIBC patients who underwent cystectomy.

### Genomic DNA Extraction and Exon Sequencing

DNA was extracted from tumor and blood samples. The tumor content was evaluated by pathologists to ensure enough tumor cells. The DNA was isolated using the DNeasy Blood and Tissue Kit (69504, QIAGEN, Venlo, Netherlands). We created targeted capture pulldown and exon-wide libraries from native DNA using the NadPrep® Hybrid Capture Reagents (for Illumina®,1005101) and NadPrep® DNA Library Preparation Kit (for Illumina®,1002103) E96, and then sequencing was performed to produce 150 bp paired end reads by using an Illumina NovaSeq platform with the average sequencing depth of 100x for controls and 200x for tumors.

### Sequence Data Quality Control

The shortreads (Raw data) were transformed from the original fluorescence image files obtained from NovaSeq platform by base calling. They were recorded in FASTQ format, including sequencing data and their corresponding quality scores. Reads with adapter contamination and low-quality/unrecognizable nucleotides were excluded through quality control. Then, downstream bioinformatics analyses were performed on clean data. At the same time, percentage of reads with average quality>20 and that of those with average quality>30, sequencing error rate, the number of total reads, and GC distribution were calculated.

### Reads Mapping and Detection of Somatic Genetic Mutation

We used the BWA (Burrows-Wheeler Aligner) (Version: 0.7.12-r1039) to align clean data to the human reference genome (hg19) and get the Sequence Alignment/Map format (*sam*) file. For the Binary Alignment/Map format (*bam*) file, the *sam* file was sorted by samtools (Version: 0.1.19-44428cd) and then used GATK (Genome Analysis Toolkit) to re-compare the reads in the interval, calibrate and rearrange the alkali matrix quality values. All bam files were used to call single nucleotide polymorphism (SNP) and insertion/deletion (Indel) by Strelka2 (version 2.9.10) (Sangtae et al., 2018). Polymorphisms of somatic single-nucleotide variants (SNVs) and InDels referenced in the 1,000 Genomes Project or Exome Aggregation Consortium (ExAC) with a minor allele frequency over 1% were removed to default filters. Those variants were then annotated by the VEP (ensembl’s Variant Effect Predictor) (version 104) software (Mclaren et al., 2016). For further analysis, FACETS (version 0.5.14), unified analysis pipeline and software were used for CNA analysis (Shen and Seshan, 2016), and GISTIC algorithm was used to infer recurrently amplified or deleted genomic regions. Based on the frequency and amplitude of amplification or deletion affecting each gene, G-scores were calculated for genomic and gene-coding regions. Genes with a significant excess of the number of non-synonymous mutations relative to the estimated density of background mutations were identified as significantly mutated genes (SMG) using the MutSigCV algorithm (Lawrence et al., 2013). Driver mutations were obtained from the “Catalog of driver mutations” curated by Integrative Onco Genomics (IntOGen) (Martínez-Jiménez et al., 2020) and The Cancer Genome Atlas project (TCGA) (Bailey et al., 2018). We focused in particular on the DDR pathway, because there were many recent reports implicating alterations in this pathway as a driver of tumor phenotype (Mouw, 2017). DDR gene list was screened by referring to published literature (Knijnenburg et al., 2018; Bellmunt et al., 2020). Mutation signature analysis was performed to resolve the SNVs for each sample into a set of characteristic patterns (signatures) to infer the contributions of each signature across samples (Alexandrov et al., 2013). All SNVs for each sample were projected onto the 30 previously described COSMIC signatures to infer the mutational signature pattern of each sample.

### Statistical Analysis

The data were expressed as medians (Q1–Q3) for the continuous variables and numbers (percentage) for the categorical variables. The Kolmogorov-Smirnov test was used to test the distribution pattern. The differences between continuous variables were determined with the Mann-Whitney U tests. The categorical variables were analyzed by χ2-test or Fisher’s exact test where appropriate. A *p*-value of less than 0.05 (two-sided) was considered statistically significant. The statistical analyses were performed with SPSS version 22.0 software (SPSS Inc., Chicago, IL, United States).

## Results

### Patient Information

The clinicopathological characteristics of enrolled patients were shown in Table 1. Among these 20 identified BCa patients, five patients were female and 15 were male, aged 32–86 years 60% (12/20) of these BCa patients were current or past smokers. Patients were divided into two groups according to pathological results: NMIBC (n = 9) and MIBC (n = 11). All NMIBC patients underwent TURBT and patholpgical stage were T1 (n = 8) or Ta (n = 1) with low grade; MIBC patients underwent laparoscopic radical cystectomy (LRC, n = 6), laparoscopic partial cystectomy (LPC, n = 4), or open partial cystectomy (OPC, n = 1), and pathological stage were T2-3 (T2a:3, T2b:4, T3a:1, T3b:3) with high grade except one case with low grade.

**TABLE 1:** Clinical information of MIBC and NMIBC patients in our study.

Category	Number	Gender	Age	T	N	M	Grade	Surgery	Smoking history
NMIBC (n = 9)	Not-IN10	F	47	1	0	0	low	TURBT	never
	Not-IN13	M	32	1	0	0	low	TURBT	never
	Not-IN14	M	62	1	0	0	low	TURBT	30 cigs/day for 30 years
	Not-IN15	M	62	1	0	0	low	TURBT	40 cigs/day for 50 years
	Not-IN16	M	67	a	0	0	low	TURBT	20 cigs/day for 40 years
	Not-IN17	F	68	1	0	0	low	TURBT	never
	Not-IN18	M	61	1	0	0	low	TURBT	20 cigs/day for 20 years
	Not-IN19	F	44	1	0	0	low	TURBT	Never
	Not-IN20	M	60	1	0	0	low	TURBT	20 cigs/day for 10 years
MIBC (n = 11)	IN1	M	75	2a	0	0	high	LRC	30 cigs/day for 50 years
	IN2	F	75	2a	0	0	high	LRC	Never
	IN3	M	79	3 b	0	0	high	LPC	10 cigs/day for 20 years
	IN4	M	66	2a	0	0	high	LPC	20 cigs/day for 44 years
	IN5	M	58	3 b	2	0	high	LRC	20 cigs/day for 30 years
	IN6	M	65	3 b	2	0	high	LRC	20 cigs/day for 40 years
	IN7	M	66	3a	0	0	high	LPC	10 cigs/day for 40 years
	IN8	M	54	2 b	0	0	high	OPC	30 cigs/day for 30 years
	IN9	M	86	2 b	0	0	high	LPC	Never
	IN11	M	64	2 b	0	0	low	LRC	Never
	IN12	F	54	2 b	0	0	high	LRC	Never

NMIBC: non-muscle invasive bladder cancer; MIBC: muscle invasive bladder cancer; F: female; M: male; TURBT: transurethral resection of the bladder tumor; LRC: laparoscopic radical cystectomy; LPC: laparoscopic partial cystectomy; OPC: open partial cystectomy.

### Mutated Driver Genes in NMIBC and MIBC Cases

**FIGURE 1:**
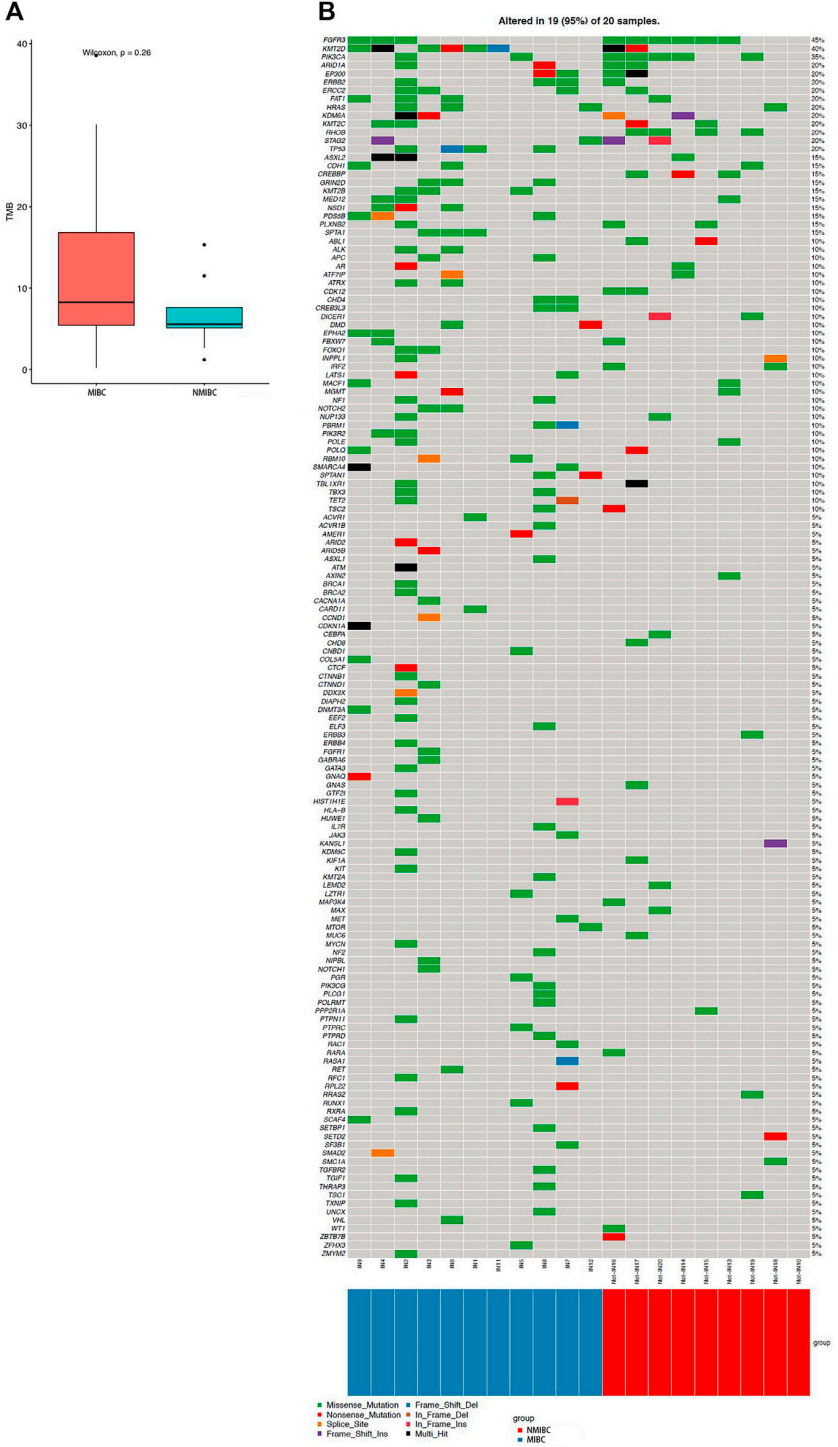
TMB and driver gene mutation profiles of bladder cancer. **(A)** TMB level in MIBC and NMIBC. Data were expressed as median (interquartile range) and were statistically analyzed by Mann-Whitney U test; **(B)** The driver gene mutation profiles in 20 bladder cancer samples. TMB: tumor mutation burden; NMIBC: non-muscle invasive bladder cancer; MIBC: muscle-invasive bladder cancer.

**FIGURE 2:**
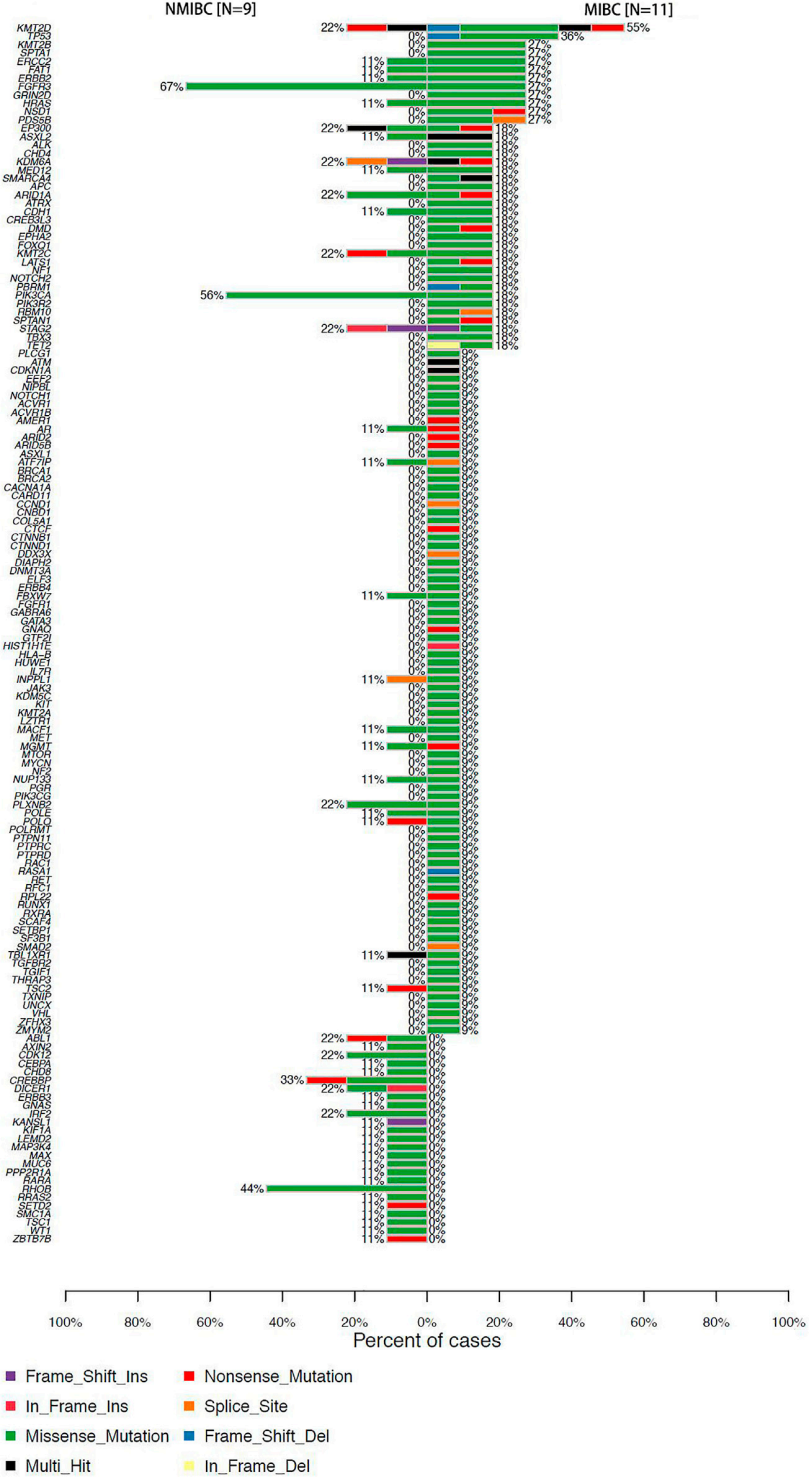
Percentages of NMIBC and MIBC cases with driver gene mutations assayed by WES. NMIBC: non-muscle invasive bladder cancer; MIBC: muscle-invasive bladder cancer; WES: whole exome sequencing.

We identified 67 SMGs in the MIBC cases, and 10 SMGs in the NMIBC cases, respectively. All detected mutations in MIBC and NMIBC were summarized in Supplementary Tables S1, S2, respectively. TMB was calculated as the number of nonsynonymous protein-coding variants divided by the total sequenced genome length. Although the difference was insignificant, the trend that MIBC had a higher TMB than NMIBC was evident [8.26 (4.63–21.84) vs 5.58 (3.87–9.58) mutations/Mb, *p* = 0.26] (Figure 1A). Next, we mainly focused on driver gene mutation profiles. Our analysis produced a final list of 281 unique driver mutations affecting 146 different genes (Figure 1B), we noticed that 95% of BCa patients (19/20) had different degrees of driver gene mutation. FGFR3 (45%), KMT2D (40%), PIK3CA (35%), ARID1A (20%), EP300 (20%), KDM6A (20%), KMT2C (20%), and STAG2 (20%) were the frequently mutated genes in BCa patients. Of note, KMT2D, KDM6A, EP300, and KMT2C were histone modification-related genes. NMIBC and MIBC displayed different genomic alterations (Figure 2). FGFR3 (67%), PIK3CA (56%), RHOB (44%), and CREBBP (33%) were the most frequently mutated genes in NMIBC patients, whereas mutations of KMT2D (55%), TP53 (36%), KMT2B (27%), SPTA1 (27%), ERCC2 (27%), FAT1(27%), ERBB2 (27%), FGFR3 (27%), GRIN2D (27%), HRAS (27%), NSD1 (27%), and PDS5B (27%) were frequently detected in MIBC. It is noteworthy that TP53, KMT2B, SPTA1, GRIN2D, NSD1, and PDS5B were only frequently mutated in MIBC but not in NMIBC patients, while RHOB and CREBBP mutations only occurred in NMIBC but not in MIBC patients. Taken together, our sequencing result indicated the prevalence of FGFR3, PIK3CA, and RHOB mutations in NMIBC and KMT2D and TP53 mutations in MIBC, which could be developed as potential biomarkers or therapeutic targets of two different types of BCa in the Chinese population.

### Somatic Mutations in Male and Female Cases

We observed Figure 1 and found that NMIMC and MIBC accounted for 60 and 40% of both sex cases, respectively (male: 9, 6/15; female: 3, 2/5), and the male and female cases displayed different genomic mutations: FGFR3 (46.7%), KMT2D (46.7%), PIK3CA (26.7%), EP300 (20%), ERBB2 (20%), FAT1 (20%), KDM6A (20%), STAG2 (20%), and TP53 (20%) were the most frequently mutated genes in male patients, whereas mutations of PIK3CA (60%), FGFR3 (40%), ARID1A (40%), ERCC2 (40%), HRAS (40%), KMT2C (40%), RHOB (40%), KMT2D (20%), EP300 (20%), ERBB2 (20%), FAT1 (20%), KDM6A (20%), STAG2 (20%), and TP53 (20%) were frequently detected in female cases. It is noteworthy that GRIN2D, PDS5B, and SPTA1 were only frequently mutated in male but not in female patients. Collectively, our sequencing result indicated the prevalence of FGFR3 and KMT2D mutations in male and PIK3CA, FGFR3, ARID1A, ERCC2, HRAS, KMT2C, and RHOB mutations in female, which needs further validation in large samples.

### DDR Gene Alterations

**FIGURE 3:**
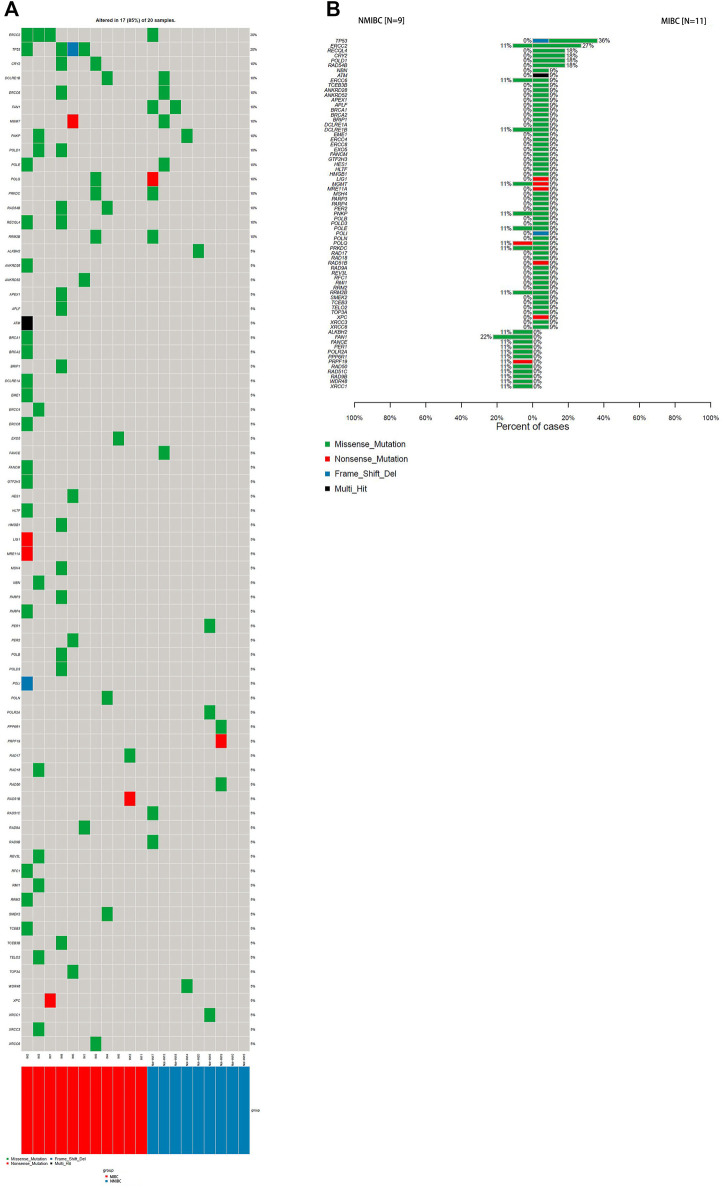
The DDR gene mutation landscape of bladder cancer samples.**(A)** WES analysis of the DDR mutated genes in 20 bladder cancer samples; **(B)** Percentages of NMIBC and MIBC cases with DDR gene mutations assayed by WES. WES: whole exome sequencing; DDR: DNA-damage repair; NMIBC: non-muscle invasive bladder cancer; MIBC: muscle-invasive bladder cancer; WES: whole exome sequencing.

It has been reported that there was an association between DDR gene mutations and responses to platinum-based chemotherapy (Teo et al., 2017) and immunotherapy treatment for BCa (Teo et al., 2018). DDR gene mutations were identified in 17 patients (85%) in this study (Figure 3A). They were seen at higher frequency in the MIBC (10/11, 91%) compared with NMIBC (7/9, 78%), besides, the average DDR mutation burden of MIBC per patient was higher than that of NMIBC (6.8 vs3.1 mutations/Mb, *p* < 0.05). ERCC2 and TP53 were the most frequently mutated DDR genes in all BCa patients, with overall mutation rates of 20 and 20%, respectively (Figure 3A). ERCC2 and TP53 variants were nearly all missense mutations (4/4, 100%; 3/4, 75%). As shown in Figure 3B, DDR gene mutations evidently occurred in MIBC patients. There were 50 DDR genes solely mutated in MIBC patients (50/71, 70%), while 12 DDR genes only mutated in NMIBC but not in MIBC patients (12/71, 17%).

### Mutation Signature Analysis

**FIGURE 4:**
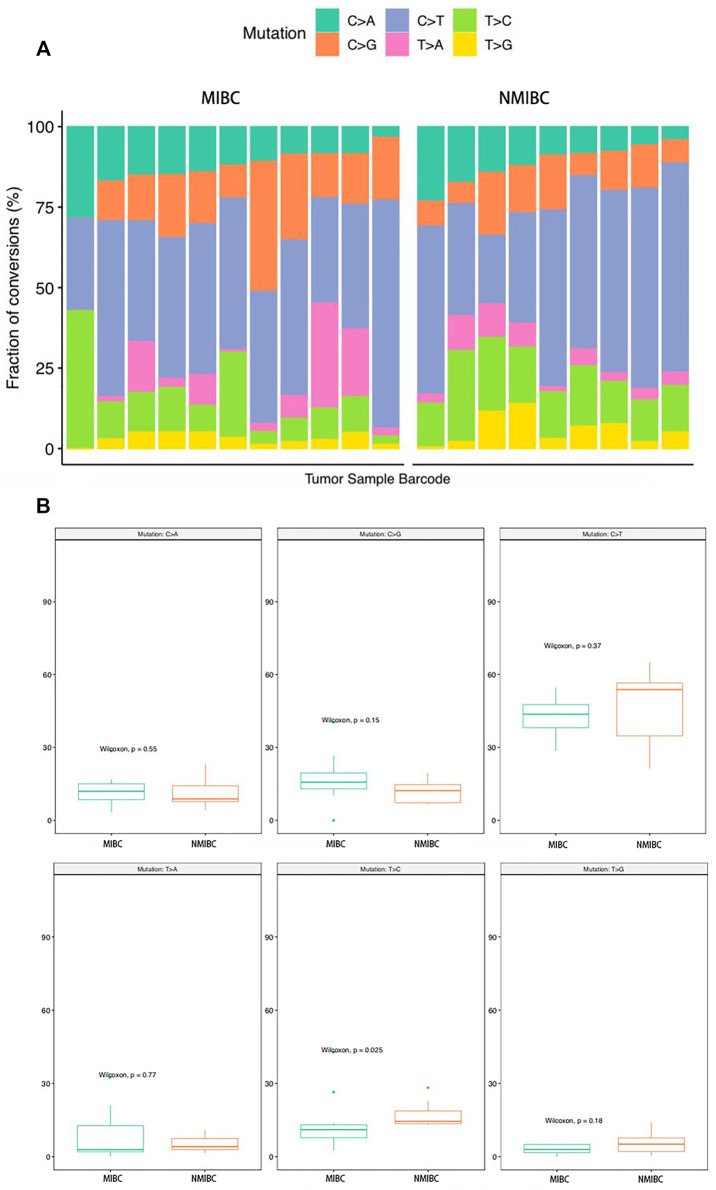
The analysis of the SNV conversions in 20 bladder cancer samples. **(A)** Fraction of all types of SNV conversions in each sample of bladder cancer; **(B)** Comparison of all SNV conversion types fractions between NMIBC and MIBC groups, data were expressed as median (interquartile range) and were statistically analyzed by Mann-Whitney U test. SNV: single-nucleotide variants; NMIBC: non-muscle invasive bladder cancer; MIBC: muscle-invasive bladder cancer.

**FIGURE 5:**
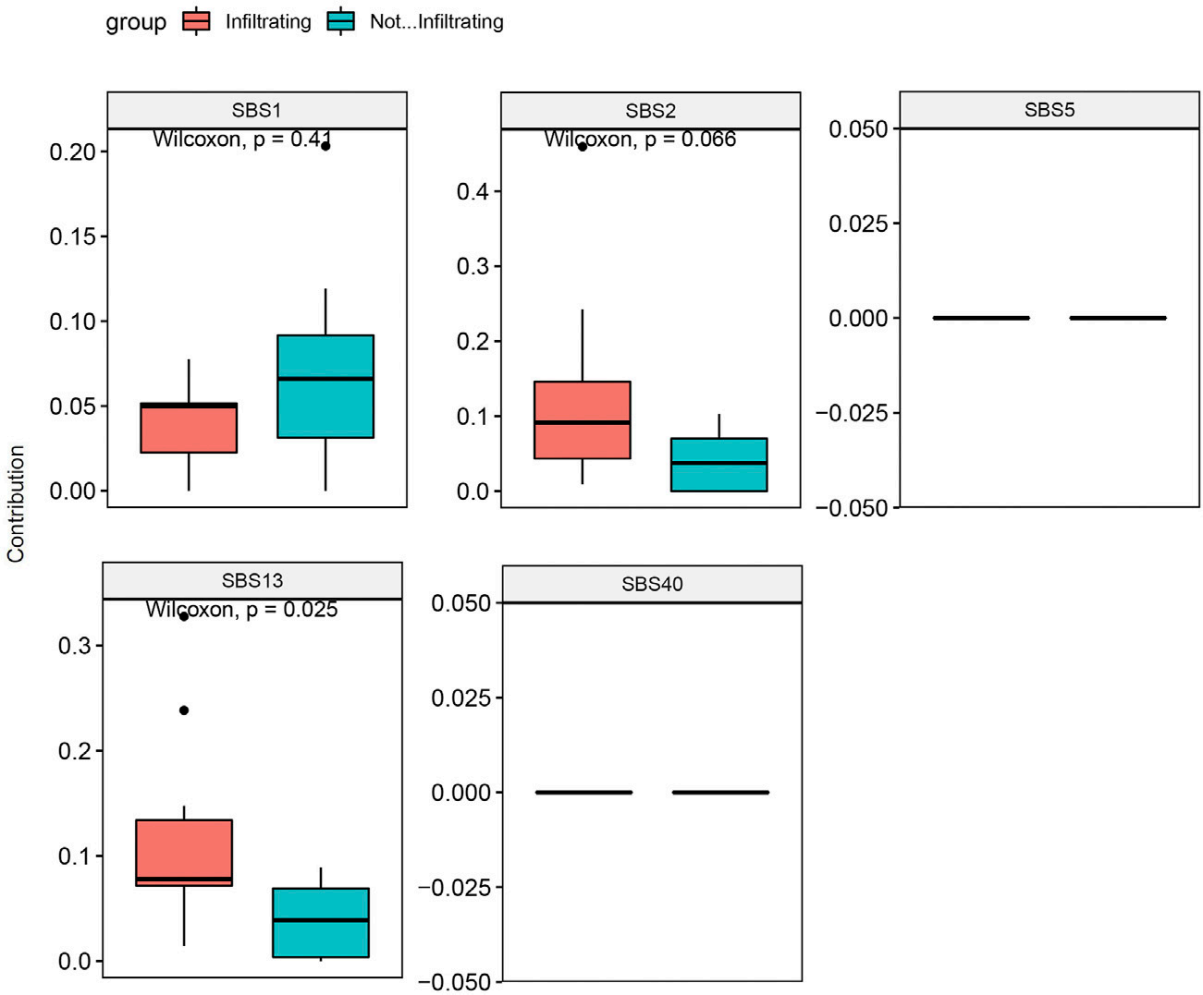
Mutation signature contribution analysis of NMIBC and MIBC samples based on SBSs 1, 2, 5, 13, and 40 for bladder cancer. Data were expressed as median (interquartile range) and were statistically analyzed by Mann-Whitney U test. NMIBC: non-muscle invasive bladder cancer; MIBC: muscle-invasive bladder cancer; SBS: single-base substitution.

SNV analysis showed that only the fraction of T > C conversion was statistically significantly higher in NMIBC than MIBC (*p* = 0.025), while the fraction of other five conversions showed no difference between the two groups (T > A, *p* = 0.77; T > G, *p* = 0.18; C > A, *p* = 0.55; C > G, *p* = 0.15; C > T, *p* = 0.37) (Figure 4A,B). Mutation signature analysis was performed to sort the SNVs for each sample into a set of characteristic patterns (signatures) to infer the contributions of each signature across samples. All SNVs for each sample were projected onto the most recent 49 single-base substitution (SBS) signatures (https://www.nature.com/articles/s41586-020-1943-3). We focused on SBS 1, 2, 5, 13, and 40, which are the main SBSs for bladder cancer (Alexandrov et al., 2020). Our analysis demonstrated that three different mutation signatures were confirmed between NMIBC and MIBC patients although the signatures exist in all BCa samples: SBS 1 (deamination of 5-methylcytosine related), SBS 2 (APOBEC related) and SBS 13 (APOBEC related) (*p* = 0.41, 0.066 and 0.025, respectively) (Figure 5). MIBC type was significantly enriched for APOBEC mutational signatures compared with NMIBC type, while NMIBC type was enriched in its SBS one signature, although not statistically significant. Collectively, these observations suggested that APOBEC mutagenesis plays important roles in the mutagenesis and evolving process of BCa, and that deamination of 5-methylcytosine related mutation was observed in a large fraction of NMIBC patients.

### Copy-Number Alterations

**FIGURE 6:**
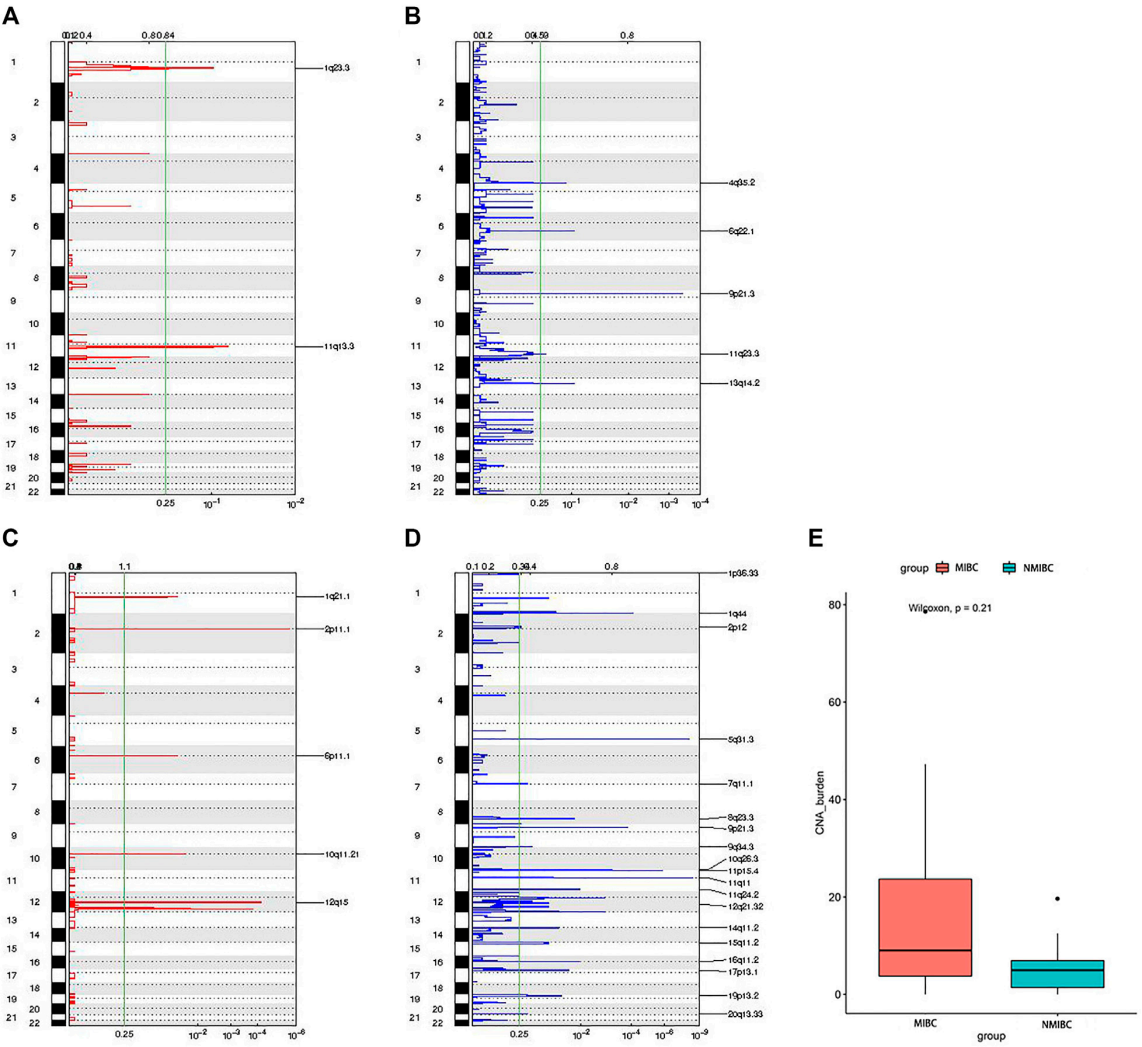
The distribution of locations of CNAs and CNAs burden between MIBC and NMIBC. **(A)** The locations of amplifications in MIBC; **(B)** The locations of deletions in MIBC; **(C)** The locations of amplifications in NMIBC; **(D)** The locations of deletions in NMIBC; **(E)** CNAs burden in MIBC and NMIBC, data were expressed as median (interquartile range) and were statistically analyzed by Mann-Whitney U test. CNA: copy number alteration; NMIBC: non-muscle invasive bladder cancer; MIBC: muscle-invasive bladder cancer.

CNAs included amplifications (amp) and deletions (del). The distribution of CNAs’ locations between MIBC and NMIBC was different. For MIBC, the locations were 1q23.3 amps, 11q13.3 amps, 4q35.2 dels, 6q22.1 dels, 9p21.3 dels, 11q23.3 dels, 13q14.2 dels (Figure 6A,B). For NMIBC, the locations were 1q21.1 amps, 2p11.1 amps, 6p11.1 amps, 10q11.21 amps, 12q15 amps, 1p36.33 dels, 1q44 dels, 2p12 dels, 5q31.3 dels, 7q11.1 dels, 8q23.3 dels, 9p21.3 dels, 9q34.3 dels, 10q26.3 dels, 11p15.4 dels, 11q11 dels, 11q 24.2 dels, 12q21.32 dels, 14q11.2 dels, 15q11.2 dels, 16q11.2 dels, 17p13.1 dels, 19p13.2 dels, 20q 13.33 dels (Figure 6C,D). It was obvious that CNAs were observed at more locations in NMIBC. However, the CNA burden of NMIBC was slightly lower than that of MIBC [4.98 (0.99–9.73) vs 9.01 (2.07–31.51) mutations/Mb] (Figure 6E).

### Gene Mutation Characteristics in Smoking and Non-smoking BCa Cases

There were 12 smokers (MIBC = 7, NMIBC = 5) and three non-smokers (MIBC = 2, NMIBC = 1) among all male cases, while all five female cases were non-smokers. In order to eliminate sex bias, we compared gene mutations between smokers and non-smokers only in men. The sequence results showed that PIK3CA (4/12), EP300 (3/12), ERBB2 (3/12), KDM6A (3/12), STAG2 (3/12), TP53 (3/12), GRIN2D (3/12), SPTA1 (3/12) were the most frequently and exclusively mutated genes in smokers when compared with non-smokers, whereas mutations of FGFR3 (2/3), KMT2D (2/3), and MACF1 (2/2) were frequently detected in non-smokers. Our data demonstrated that there was an evident trend that the average driver gene mutations of smokers per patient was higher than that of non-smokers (12 (7–17) vs 7 (1–14) mutations/Mb, *p* = 0.24), especially in MIBC subgroup (16 (11.5-21.3) vs 7.5 (1–14) mutations/Mb, *p* = 0.25) (Supplementary Figure S1A,B). The average DDR mutation burden of smokers per patient was marginally higher than that of non-smokers [4.5 (1.8-11.3) vs 2.5 (0–5) mutations/Mb, *p* = 0.46] in MIBC subgroup (Supplementary Figure S1C). Besides, our results showed an evident trend that smokers had higher TMB burden than non-smokers [7.6 (5.6-15.3) vs 5.8 (0.2-10.1) mutations/Mb, *p* = 0.37], especially in MIBC subgroup [10.0 (6.8-23.9) vs 5.2 (0.2-10.1) mutations/Mb, *p* = 0.43] (Supplementary Figure S1D,E). Moreover, there was a trend that the average CNA burden of smokers was higher than that of non-smokers [31.5 (8.0-70.8) vs 20.3 (9.0-31.5) mutations/Mb, *p* = 0.80] in MIBC subgroup (Supplementary Figure S1F). Collectively, there was a trend that smokers had higher driver gene and DDR mutation frequency, higher TMB and CNA burden than non-smokers in BCa patients, and this trend was more evident in MIBC cases.

## Discussion

The present study was the first to comparatively analyze differentially expressed genes in NMIBC and MIBC in the Chinese population by WES. Beyond confirming mutated genes previously identified in BCa such as FGFR3, KMT2D, PIK3CA, ARID1A, EP300, KDM6A, KMT2C, and STAG2, we identified additional driver gene mutations that were implicated in NMIBC and MIBC. Notably, we discovered that TP53, KMT2B, SPTA1, GRIN2D, NSD1, and PDS5B were only frequently mutated in MIBC patients, while RHOB and CREBBP mutation only occurred in NMIBC. Furthermore, we firstly detected five mutation signatures differently expressed between NMIBC and MIBC patients. The current study established a profile of variation in gene mutations between NMIBC and MIBC, which might have potential clinical implications with regard to providing novel targets for treatment and exploring potential biomarkers to distinguish these two types of BCa.

We identified 77 SMGs in the 20 BCa cases (67 in MIBC, and 10 in NMIBC). In our study, we demonstrated that FGFR3 (45%), KMT2D (40%), PIK3CA (35%), ARID1A (20%), EP300 (20%), KDM6A (20%), KMT2C (20%), STAG2 (20%) and TP53 (20%) were the most frequently mutated genes in BCa patients, which included three well-known BCa genes (TP53 (Cordon-Cardo et al., 1994), FGFR3 (Jebar et al., 2005), PIK3CA (Platt et al., 2009)). Data from TCGA Research Network showed that TP53 (49%), MLL2 (27%), ARID1A (25%), KDM6A (24%), PIK3CA (20%), EP300 (15%), CDKN1A (14%), RB1 (13%), ERCC2(12%), FGFR3 (12%), STAG2 (11%) were the most frequently mutated genes in BCa (The Cancer Genome Atlas Research Network, 2014). Data of 103 China BCa samples downloaded from International Cancer Genome Consortium (ICGC) database showed that TP53 (27.2%), KDM6A (24.3%), FGFR3 (16.5%), HRAS (15.5%), ERBB2 (13.6%), CREBBP (12.6%), ERCC2 (12.6%), ARID1A (11.7%), and XIRP2 (10.7%) were the top mutated genes (Wang et al., 2020). One recent study conducted by Wang T et al. performed targeted NGS in 32 BCa samples showed that FGFR3 (31.3%), KMT2D (18.8%), TP53 (12.5%), ARID1A (12.5%), AKT1 (12.5%), KDM6A (9.4%), STAG2 (9.4%), and LRP1B (9.4%) were the most frequently mutated genes in Chinese BCa patients (Wang et al., 2020). Wu S. et al. (Wu et al., 2019) found FGFR3, TP53, PIK3CA, ZFP36L1, HRAS, and KDM6A were frequently mutated in Chinese BCa patients. Generally speaking, these previous investigations are consistent with our results and complement one another.

In addition, we observed frequent mutations in chromatin-remodeling genes, including the histone demethylase gene KDM6A (NMIBC 22%; MIBC 18%), chromatin-remodeler gene ARID1A (NMIBC 22%; MIBC 18%), histone lysine methyltransferase genes KMT2B (NMIBC 0%; MIBC 27%), KMT2C (NMIBC 22%; MIBC 18%), KMT2D (NMIBC 22%; MIBC 55%), and the histone acetyltransferase gene EP300 (NMIBC 22%; MIBC 18%). These somatic mutations in chromatin-remodeling genes indicated that altered epigenetic regulation of chromatin and post-translational modifications might be a major driver mechanism in BCa. Gui Y, et al. identified genetic aberrations of the chromatin remodeling genes (including EP300 and ARID1A) in 59% of the 97 subjects with MIBC (Gui et al., 2011). Besides, KDM6A (Chen et al., 2021), KMT2C (Baker et al., 2020; Hirotsu et al., 2020), and KMT2D (Baker et al., 2020; Baker et al., 2021) were believed to play important roles in bladder carcinogenesis and attracted more attention in the recent years. However, there was no report on the relationship between KMT2B mutation and BCa. Our study was the first to demonstrate that KMT2B mutation occurred in 27% of MIBC only, not in NMIBC, suggesting that KMT2B might trigger the evolution from NMIBC to MIBC.

It is well known that NMIBC often harbor mutations in FGFR3 and the Ras gene family, and MIBC usually have defects in TP53 and RB1 in high-grade tumors (Gui et al., 2011), which further confirmed our findings. It was noteworthy that several mutations (TP53, KMT2B, SPTA1, GRIN2D, NSD1, and PDS5B) were only found in MIBC but not in NMIBC patients. It is noteworthy that TP53 mutation frequency in MIBC was 36.4% (4/11) in our study. We also analyzed the sequencing data of 103 Chinese BCa samples download from ICGC database and found that TP53 mutation frequency was 26.3% (10/38) for NMIBC, and 27.7% (18/65) for MIBC. Targeted NGS results given by Wang T et al. showed that TP53 mutation frequency was 25% (4/16) for NMIBC, and 0% (0/16) for MIBC in 32 Chinese BCa patients (Wang et al., 2020). Meanwhile, we also checked frequently mutated genes in BCa samples from TCGA cohort, and identified that TP53 mutation frequency was 46.96% (193/411) for MIBC. It is evident that the prevalence of TP53 mutations in Chinese Han is much lower than that in Europeans. The varied distribution of this important tumor suppressor may be due to ethnic differences.

In addition to TP53, the above identified mutated genes specific to MIBC should be further investigated to clarify the underlying mechanisms responsible for MIBC. Previous studies paid less attention on genetic mutations in NMIBC, and our results showed that FGFR3 (67%), PIK3CA (56%), RHOB (44%), and CREBBP (33%) were the top mutated genes in NMIBC. As FGFR3 and PIK3CA mutations were also found in MIBC, but at a low frequency, we speculated that these tumors progressed from NMIBC. More importantly, we found mutations in the Ras superfamily GTPase member RHOB only occurred in NMIBC but not in MIBC patients, suggesting that these NMIBC with RHOB mutation may not progress to MIBC. Moreover, a combination of RHOB and other frequently mutated genes could form a multi-gene panel utilized for NMIBC diagnosis and risk stratification.

As for the comparison of somatic mutations between male and female BCa cases, we found that both PIK3CA and FGFR3 were the top mutated genes in both sexes. The KMT2D mutation was significantly more common in males than in females (46.7 vs 20%).

KMT2D is located at 12q13.12 (Froimchuk et al., 2017) and encodes a histone H3 lysine 4 (H3K4)-specific methyl transferase (Bögershausen et al., 2016). KMT2D mutation may partly explain the different BCa incidences of men and women in China (M: F = 3.4:1) (Pang et al., 2016). Besides, our data showed that KDM6A mutation frequency in males was the same as in females (20%). KDM6A is located at Xp11.3, encoding a histone H3K27-specific demethylase and escapes X chromosome inactivation (Greenfield et al., 1998; Hong et al., 2007). It is reported that KDM6A mutations were more common in females than males in NMIBC (Hurst et al., 2017). However, our data did not show that trend, which could be attributed to small sample size. Although men’s bladder cancer is more common, women’s prognosis is worse even after environmental factors have been corrected (Nakayama et al., 2019). Although this is due to the difference in hormones between men and women, it may also be the result of the protective effects of two functional copies of KDM6A in females.

DDR mutation has long been implicated in both carcinogenesis and prognosis of urothelial carcinoma. Polymorphisms in various DDR genes such as ERCC2 have previously been associated with the development of urothelial carcinoma (Stern et al., 2009). Our study found that DDR gene mutations such as ERCC2 and TP53 were seen at higher frequency in MIBC than NMIBC (91 vs 78%). It was reported that there was a strong positive correlation between DDR gene mutation and TMB in urothelial carcinoma (Nassar et al., 2019). Our results showed an evident trend that MIBC had a higher TMB than NMIBC, which confirmed the association between DDR mutation and TMB. We supposed that DDR gene mutation set forth a driver for mutations, or maybe high TMB reflects more opportunity for mutation in the set of DDR genes.

Smoking is a significant modification risk for BCa, and almost half of men and one-quarter of women with BCa are believed to be attributed to smoking (Ploeg et al., 2009). Smoking may cause several genetic mutations, such as destroying DNA and reducing repair activity (Lee et al., 2018). Our data indicated that there was a trend that smokers had higher DDR mutation frequency and TMB or CNA burden than non-smokers in BCa especially MIBC patients, although not statistically significant which may be caused by small sample size. Our sequencing results showed that mutations of important DDR gene (including TP53, ERCC2) and driver gene (including KDM6A, SPTA1) occurred frequently in smokers rather than non-smokers, which was consisted with the previous research (Hayashi et al., 2020). We assume that smoking may firstly cause DDR gene mutation and the loss of repair function further causes driver gene mutation and more TMB.

To examine the mutation spectrum, we applied nonnegative matrix factorization and identified three different mutation signatures between NMIBC and MIBC patients in the main SBSs for bladder cancer: MIBC (SBS 2, 13); NMIBC (SBS 1). Our study showed that the main endogenous mutation signature in MIBC was SBS 2 and 13 (attributed to APOBEC enzyme activity), which, coupled with the process of DNA repair, yielded both C-to-T and C-to-G mutations (Roberts et al., 2013). APOBEC enzymes were reported to be part of the innate antiviral immunity and cancer mutagenesis (Swanton et al., 2015). SBS 1, a clock-like mutational signature, is closely associated with age at diagnosis and persisted throughout the patient’s life (Jiang et al., 2021). There was a trend that SBS1 occurred more frequently in NMIBC in our study, indicating SBS1 played important roles in the triggering step of BCa carcinogenesis. Furthermore, our data indicated that SBS 2 and 13 could be potential therapeutic targets for the treatment of BCa, because these signatures were positively correlated with stage and tumor burden, suggesting they may be involved in the progression of BCa. Moreover, our results showed that CNAs occurred at more locations on chromosomes in NMIBC than MIBC, suggesting that NMIBC and MIBC might develop secondary to different molecular mutations.

## Data Availability

The data presented in the study are deposited in the S R A repository, accession number SUB11071815, https://www.ncbi.nlm.nih.gov/Traces/study/?acc=PRJNA805388.

## Supplementary Material

Supplementary Figure S1 The analysis of driver gene and DDR mutation, and TMB and CNA burden in smoking and non-smoking male bladder cancer cases.**(A)** Comparison of driver gene mutation between smokers and non-smokers; **(B)** Comparison of driver gene mutation between smokers and non-smokers in MIBC patients; **(C)** Comparison of DDR mutation between smokers and non-smokers in MIBC patients; **(D)** Comparison of TMB between smokers and non-smokers; **(E)** Comparison of TMB between smokers and non-smokers in MIBC patients; **(F)** Comparison of CNA burden between smokers and non-smokers in MIBC patients, data were expressed as median (interquartile range) and were statistically analyzed by Mann-Whitney U test. DDR: DNA-damage repair; TMB: tumor mutation burden; CNA: copy number alterations; NMIBC: non-muscle invasive bladder cancer; MIBC: muscle-invasive bladder cancer; ns: not significant.
